# Lumbopelvic pain and sick leave during pregnancy: A comparison of Italy and Norway

**DOI:** 10.1177/17455057231218197

**Published:** 2023-12-11

**Authors:** Lene Annette Hagen Haakstad, Maria Beatrice Benvenuti, Emilie Mass Dalhaug, Kari Bø

**Affiliations:** 1 Department of Sports Medicine, Norwegian School of Sport Sciences, Oslo, Norway; 2 Department of Obstetrics and Gynecology, Akershus University Hospital, Nordbyhagen, Norway

**Keywords:** between-country comparison, pelvic girdle pain, pregnancy, severity, sick leave

## Abstract

**Background:**

Pregnancy-related lumbopelvic pain is a frequently reported musculoskeletal disorder, but few studies have compared data between countries.

**Objectives:**

Examine prevalence, severity, and sick leave and explore potential risk factors associated with pregnancy-related lumbopelvic pain in Italian women and compare the results to a similar study in Norway, utilizing the same questionnaire.

**Design:**

Cross-sectional

**Methods:**

Italian (*n* = 481) and Norwegian women (*n* = 435) were allocated from two public hospitals in Rome (Fatebenefratelli San Giovanni Calibita-Isola Tiberina) and Oslo (Oslo University Hospital), as well as four antenatal clinics in Modena (Italy). The questionnaire was completed between gestation weeks 32 and 36, addressing women’s experiences of pregnancy-related lumbopelvic pain and sick leave in current week, and retrospectively for prepregnancy, first and second trimesters.

**Results:**

In Italy and Norway, 39% and 57% of pregnant women reported pregnancy-related lumbopelvic pain, respectively, with 11% and 25% experiencing severe pregnancy-related lumbopelvic pain. Pregnancy-related lumbopelvic pain was associated with sick leave in Norway (*p* < 0.01), but not in Italy (*p* = 0.66) at late gestation. In both countries, women with pregnancy-related lumbopelvic pain versus those with no pregnancy-related lumbopelvic pain were more likely to be multiparous (Italy: 40% versus 31%, *p* = 0.06 and Norway: 53% versus 38%, *p* < 0.01), and have gestational weight gain above guidelines (Italy: 21% versus 13%, *p* = 0.02% and Norway: 27% versus 14%, *p* < 0.01) and previous experience of pregnancy-related lumbopelvic pain (Italy: 15% versus 2%, *p* < 0.01 and Norway: 31% versus 4%, *p* < 0.01). Maternal exercise (⩾2 times weekly) was associated with less pregnancy-related lumbopelvic pain (Italy: odds ratio = 0.33, 95% confidence interval = 0.11–1.0, *p* = 0.05 and Norway: odds ratio = 0.55, 95% confidence interval = 0.29–1.0, *p* = 0.06).

**Conclusion:**

We observed high rates of pregnancy-related lumbopelvic pain in Italy and Norway, with Norwegian women reporting the highest prevalence and severity level. While both countries had similar rates of sick leave in late gestation, an association between pregnancy-related lumbopelvic pain and sick leave was observed among Norwegian women only. Health care providers should be proactive in addressing pregnancy-related lumbopelvic pain through open communication and seeking input from pregnant individuals. However, it is essential to acknowledge that the current evidence on effective treatments remains limited and inconclusive, highlighting the need for further research in this field.

## Introduction

Pregnancy is accompanied by hormonal, biomechanical, and physiological changes that may result in increased body mass and ligament laxity, as well as decreased abdominal muscle strength.^1,2^ These bodily alterations become more prominent as pregnancy progresses and shift the center of gravity.^1^ Although postural changes during pregnancy can contribute to pelvic girdle pain (PGP) and low back pain (LBP), it is important to recognize that pain in the lumbopelvic area is multifactorial and may extend beyond mere biomechanics. Hence, it must be viewed with the evolution of contemporary pain science, which acknowledges biopsychosocial factors.^3–5^ PGP is one of the most common musculoskeletal complaint in pregnancy and may be experienced isolated or together with LBP.^2,6,7^ The European guidelines^2^ describes PGP as pain experienced between the posterior iliac crest and the gluteal fold and/or in the pubic symphysis, whereas LBP is pain localized below the ribs, but above the gluteal folds, with or without radiation down the legs.^8^ The term pregnancy-related lumbopelvic pain (PLPP) is often used when no distinction is made between PGP and LBP. This type of pain can range from a mild complaint to a more severe pain, affecting daily activities, physical and psychological quality of life.^8^ The etiology, however, remains poorly understood, and PLPP is often treated from a purely biomechanical perspective.^5^ The number of women with PLPP (4%–86%) and severe PLPP (20%–54%) differs greatly between studies depending on definitions and measurement methods used to diagnose the condition.^9–13^

Even though PLPP is found to be a common complaint in most studies (50%), linked to decreased health-related quality of life (HRQoL) and the pregnant women’s workability, it has been found to be overlooked in some countries.^7,8,10,14^ For instance, it is discussed whether PLPP is a complaint during pregnancy that must be tolerated, or whether it is a major public health issue and a reason for prescribing sick leave.^15–17^ In Scandinavia, PLPP and PGP account for most of the sick leave in pregnancy with an average length of 12–15 weeks^16,18,19^ and, therefore, have considerable socioeconomic implications.^18^ It is also a general belief that PLPP is more prevalent in these countries (Norway, Sweden, and Denmark), but this is probably related to the large number of studies conducted in these countries.^15,16,18–23^ In addition, it has been suggested that the percentage of sick leave may be related to the social welfare system and cultural beliefs. However, the few studies that have compared ethnicity and geographical setting do not differ in perceived PLPP during pregnancy.^10,15,24^

Worldwide, and even within several European countries, there is a distinct lack of studies examining PLPP. Unlike the Scandinavian countries, pregnancy and motherhood has a strong traditional role in Italy, including the role of “la famiglia” (the family), being the fundamental social institution, based on mutual aid for all family members. This ideology might impact the attitudes of the women and the family, and result in less awareness of the impairment caused by pregnancy complaints in general, including PLPP. Also, a systematic search on PubMed.gov received no results on PLPP in an Italian population. Hence, the primary aims of this study were to examine the prevalence, severity, and self-reported sick leave and explore potential risk factors associated with PLPP in pregnant Italian women. In addition, we wanted to compare the results with those from a corresponding study conducted in Norway, utilizing the same questionnaire.

## Methods

This transnational comparison project was designed as a cross-sectional study with a self-administered questionnaire, completed by a total of 916 women; *n* = 481 from Italy (2018) and *n* = 435 from Norway (2005). Women were recruited to the study from two public hospitals in Rome (Fatebenefratelli San Giovanni Calibita-Isola Tiberina) and Oslo (Oslo University Hospital, Rikshospitalet), as well as four antenatal clinics in Modena (Italy).

### Participants and enrollment

**Figure 1.**
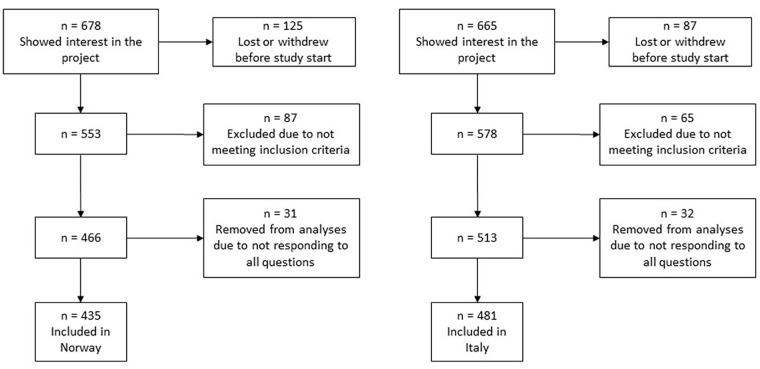
Flowchart illustrating the recruitment process in Norway and Italy.

In Norway, women were enrolled through the application form for birthing services at Rikshospitalet, Oslo University Hospital. Women are advised to initiate the application process early in pregnancy to ensure timely access to appropriate prenatal care and to secure a place for delivery. As a result, most women were recruited in early second trimester. In Italy, the women were approached by one investigator during a routine appointment with the medical staff at gestation weeks 30–32. The questionnaire, including assessments of the number of women experiencing PLPP, was completed either at the clinic or at home between gestational weeks 32–36. Due to this timeline, some participants who initially expressed interest in the study were lost or withdrew before study start and data collection (Italy: 87 women and Norway: 125 women). General inclusion criteria in both countries were age ⩾18 years, being a permanent resident of either a Scandinavian country or Italy, and being able to respond to the questionnaire, including questions about PLPP in the third trimester (gestational weeks 32–36). Criteria for exclusion were multiple pregnancy and reported risks for adverse pregnancy outcomes or fetal pathologies (i.e. hypertension, pre-eclampsia, gestational diabetes, premature contractions, intrauterine growth restriction, and placental abnormalities). Nulliparous was defined as a woman expecting her first child and multiparous as a woman who had given birth to one or more children before. After having applied all the exclusion criteria, we were left with 513 (88.8%) of 578 Italians and 466 (84.3%) of 553 Norwegians that signed an informed written consent and completed assessments. Only women responding to all questions were included in the analyses. Hence, 32 and 31 responders from Italy and Norway were removed from the present analyses, giving a final response rate of 83.2% and 78.7%, respectively. A flowchart illustrating the recruitment process is presented in Figure 1.

### Sample size and representativeness considerations

The sample size for this study was determined based on various factors. At Rikshospitalet, Oslo University Hospital, about 2000 women give birth annually. However, due to logistical limitations and that the initial study included several investigations and outcome parameters beyond the scope of assessing proportions reporting PLPP, it was not feasible to approach all eligible women. Unfortunately, no power calculation was conducted for the assessment of PLPP, self-reported sick leave, and potential risk factors. Between 2003 and 2005, we invited a total of 2145 women to participate in the study, including approximately one-third of those giving birth during that period. A proportion slightly exceeding one-fourth of the invited women accepted the invitation. To ensure a similar sample size for the Italian group, we aimed to attain a comparable number of participants to the Rikshospitalet University Hospital group, facilitating meaningful comparative analyses between the Norwegian and Italian women.

To assess the representativeness of the participants in terms of a general urban pregnant population in Norway, a comparison analysis was performed with data from 150 nonparticipants who gave birth at the same hospital. No differences were found concerning maternal age, parity, marital status, and educational level.^25^ No such analysis was done for the Italian participants due to limitations in the hospital electronic database structure, which did not allow for a direct comparison.

### Assessment procedures and outcome measures

The trimester-specific Physical Activity and Pregnancy Questionnaire (PAPQ) was used to obtain information on demographic, health, and lifestyle characteristics (including proportions of daily smokers and previous smokers), pregnancy complaints and PLPP, as well as reports of being sick-listed.^26^ Assessments of the number of women experiencing PLPP included a yes or no response to the question: *“During this pregnancy, do you experience pain in the lumbopelvic area?”* This approach allowed us to capture the overall prevalence of PLPP, encompassing both isolated PGP and PGP co-existing with LBP. For those who responded positively, we further investigated the degree of disability or severity by asking: *“Do you have problems walking to the extent of using crutches?”* The response options were as follows: *“Not at all,” “Occasionally,” “Sometimes,” or “Most of the day.”* Severe PLPP was defined as using crutches (responding to one of the three latter options). The two questions on PLPP were asked cross-sectionally at gestational weeks 35.9 ± 2.0 (Italy) and 36.4 ± 1.7 (Norway), and posed retrospectively for first and second trimesters. Furthermore, we included two questions to obtain information about foregoing PLPP, as this is shown to be an important risk factor when examining PLPP at present.^2,7^ *“During previous pregnancies, did you experience any lumbopelvic pain?”* (*“Yes” or “No”).* A yes response was followed by a question investigating the duration of the pain period postpartum: *“When did the pain in the pelvic area stop?”* The response options were as follows: *“Less than 6 weeks after delivery,” “6–20 weeks after delivery,” “5–10 months after delivery,” or “persistent pain.”*

Pregnant employees in Italy and Norway are entitled to paid sick leave for medical reasons.^27,28^ In this study, participants provided self-reported sick leave data for the first and second trimesters retrospectively, while data for third trimester were collected prospectively, reflecting their current status. Responses were given as *“no sick leave”* or *“full-time sick leave.”* We did not ask about part-time sick leave (percentage) or primary cause of pregnancy-related sick leave, but combined available information and created two groups: *“women who reported PLPP and were on sick leave during pregnancy”* and *“women who reported PLPP and were not on sick leave during pregnancy.”*

Frequency of recreational exercise/sport prepregnancy and in the third trimester (moderate intensity leisure-time physical activity ⩾20 min) was part of the health and lifestyle section in the questionnaire, assessed by six response alternatives: *“⩽1 per week,” “2–3 times per week,” “4–5 times per week,” “6 times per week,”* and *“every day or more than once every day.”* Following Malmqvist et al.^19^ being a regular exerciser was defined as performing moderate intensity (light breathing and modest sweating) leisure-time physical activity ⩾2 times a week.

To assess occupational workload, we included two different questions with the following definitions: *“Would you characterize your paid work as physically demanding?”* (*“Yes”* or *“No”*). *“Do you usually perform your work standing and/or walking?”* (*“>50% of the time”*).

Calculation of prepregnancy body mass index (BMI) was based on self-reported weight (kg) and height (m), using the following formula: BMI = weight (kg) / height^2^ (m^2^). The cutoff points were grouped according to World Health Organization (WHO) BMI classification: underweight (<18.5 kg/m^2^), normal weight (18.5 to 24.9 kg/m^2^), overweight (25 to 29.9 kg/m^2^), and obese (⩾30 kg/m^2^).^28^ Gestational weight gain (GWG) was reported by the participants and further divided into GWG categories, using prepregnancy BMI groupings recommended by the Institute of Medicine (IOM).^29^

### Rationale for our paper-based survey approach

Before 2010, paper surveys were the most common method of collecting data, and this was the method we used for the Norwegian participants in 2005. Thus, to have a similar approach, this was also done in Italy in 2018. To achieve high cross-language validation when translating the Norwegian version of the PAPQ into Italian, we used a forward–backward translation technique, involving a bilingual Italian research assistant with Italian as native language.^29^ Based on this, some adjustments were made. A pilot test of the Italian version of the PAPQ including 10 pregnant women led to minor changes in format, layout, and wording.

### Statistical analysis

All statistics were conducted with Statistical Package for the Social Sciences (SPSS) Software V. 24 for Windows. Descriptive data were screened for normality and outliers, including a comparison of the overall curve of the bars of the histograms, and the usage of parametric statistics. Chi-squared analyses were used to compare categorical data, and a two-sided independent sample *t*-test was used for continuous data. The selection of variables for the multivariable logistic regression was based on the cited literature^7^ and by performing univariable logistic regression. This approach allowed us to identify variables that showed significant associations or demonstrated clinical relevance. Subsequently, multiple logistic regression, separately for Italian and Norwegian women, were used to investigate the associations between PLPP (dichotomous-dependent variable, coded 0/1) and the predictive power of several selected variables: educational level, parity, prepregnancy BMI (kg/m^2^), GWG, previous PLPP, occupational workload, and exercise prepregnancy and at present.^7^ Through these analyses, we explored the contribution of several factors while accounting for their potential confounding role. In a similar vein, we used a logistic regression model including the same factors to investigate their contribution to sick leave due to PLPP. For the Italian group, a low number of women self-reported sick leave and PLPP (*n* = 52) in the second trimester and the multiple logistic regression was limited to five factors (parity, BMI, GWG, previous PLPP, and maternal exercise). All variables were categorized, and one specific category was designated as the reference group. The choice of the reference group was based on the research question, and the characteristics of the variables analyzed. For example, when considering the factor multiparous, the reference group was set as the category nulliparous. Likewise, for the variable regular maternal exercise, the reference group was set as the category no exercise. This approach allowed for meaningful comparisons and facilitated the statistical interpretation. The results are presented as frequencies (*n*) and percentages or mean with standard deviation (*SD*), as well as group differences and odds ratio (OR) with 95% confidence interval (CI) and *p* values. The level of statistical significance was set at *p* ⩽ 0.05.

## Results

At the time of data collection, there was some discrepancy between the participants, with more Italians being nulliparous, older, less educated, fewer were overweight or obese (BMI ⩾25), had gained weight above the IOM guidelines,^30^ and were exercising regularly compared with the Norwegian participants (Table 1).

**Table 1.** Chi-squared analyses (categorical data) and two-sided independent sample *t*-test (continuous data) comparing demographic and health characteristics of the Italian and Norwegian participants (*n* = 916).

Variable	Italy (*n* = 481)	Norway (*n* = 435)	Group difference (95% CI)	*p*
Age (year), *M* (SD)	34.4 (5.4)	31.6 (4.1)	2.8 (2.2 to 3.4)	<0.01
Parity, *n* (%)				
– Multiparous	167 (34.7)	214 (49.2)	14.5 (8.1 to 20.7)	<0.01
– Children, *M* (*SD*)	1.3 (0.7)	1.3 (0.5)	—	—
Cohabitation/married, *n* (%)	465 (96.7)	430 (98.9)	2.2 (0.2 to 4.3)	0.03
Education, *n* (%)				
– No higher education	209 (43.5)	74 (17.0)	28.3 (22.5 to 33.8)	<0.01
– University/college degree	272 (56.5)	361 (83.0)	26.5 (20.7 to 32.0)	<0.01
Employment outside home, *n* (%)				
– ⩾50% (third trimester)	123 (25.6)	211 (48.5)	22.9 (16.7 to 28.9)	<0.01
Tobacco use, *n* (%)				
– Daily smoker	38 (7.9)	12 (2.8)	5.1 (2.2 to 8.1)	<0.01
– Previous smoker	175 (36.4)	165 (37.9)	1.5 (−4.7 to 7.7)	0.64
Prepregnancy BMI (kg/m^2^), *M* (*SD*)	22.8 (3.9)	23.5 (3.8)	0.7 (0.2 to 1.2)	<0.01
Prepregnancy BMI ⩾25 (kg/m^2^), *n* (%)	93 (19.3)	132 (30.3)	11.0 (5.4 to 16.5)	<0.01
GWG (kg), *M* (*SD*)	12.0 (4.2)	13.2 (4.6)	1.2 (0.6 to 1.8)	<0.01
GWG above IOM^a^ guideline, *n* (%)	83 (17.3)	94 (21.6)	4.3 (−0.8 to 9.5)	0.10
Regular exercise/sport^b^, *n* (%)				
– Prepregnancy	196 (40.7)	378 (86.9)	46.2 (40.5 to 51.4)	<0.01
– At present (third trimester)	89 (18.5)	216 (49.7)	31.2 (25.2 to 36.9)	<0.01
Sick leave, *n* (%)				
– First trimester	65 (13.5)	27 (6.2)	7.3 (3.5 to 11.1)	<0.01
– Second trimester	103 (21.4)	53 (12.2)	9.2 (4.4 to 14.0)	<0.01
– Third trimester	186 (38.7)	150 (34.5)	4.2 (−2.0 to 10.4)	0.19

CI: confidence interval; *SD*: standard deviation; BMI: body mass index; GWG: gestational weight gain; IOM: Institute of Medicine; *M*: mean.

a Gaining above was defined as upper limit of IOM range for corresponding prepregnancy BMI group (18 kg for underweight, 16 kg for normal weight, 11.5 kg for overweight, and 9 kg for obese).

b Moderate intensity (light breathing and modest sweating) leisure-time physical activity ⩾2 times weekly.

### Prevalence, severity, and self-reported sick leave of PLPP in Italy and Norway

In Italy, 39.1% reported having experienced PLPP in the present pregnancy and 10.6% suffered from severe PLPP in the third trimester. The corresponding numbers in Norway were 56.6% and 24.8%, respectively. Compared with Norwegian women, a higher percentage of Italian women utilized sick leave during the first and second trimesters, while a similar proportion of both groups utilized sick leave during late gestation (Table 1). Prevalence, severity, and duration of PLPP are shown in Table 2. Experiencing PLPP was associated with self-reported sick leave in the second trimester among the Italian women, and in the second and third trimester among the Norwegian women (Table 3). In the subanalyses of the Italian group, 75 out of 188 reported PLPP and being on sick leave (27.7%), but none of the factors in the model (parity, prepregnancy BMI, GWG, previous experience of PLPP, and regular exercise) were associated with sick leave in the second trimester. In the Norwegian group, 102 out of 242 women reported PLPP and being on sick leave at late gestation (42.1%), and three factors were significantly associated: previous experience of PLPP and severe PLPP gave higher OR, whereas regular maternal exercise a lower OR. More details of prevalence and relevant factors (background, health, lifestyle, and self-reported sick leave) associated with PLPP are shown in Tables 3 and 4.

**Table 2.** Chi-squared analyses comparing prevalence, severity, and duration of PLPP by country (*n* = 916).

Variable	Italy (*n* = 481)	Norway (*n* = 435)	Group difference (95% CI)	*p*
PLPP, *n* (%)	188 (39.1)	242 (56.6)	17.5 (11.0 to 23.8)	<0.01
Severe PLPP, *n* (%)				
– First trimester (weeks 1–12)	11 (5.9)	31 (12.8)	6.9 (1.2 to 12.3)	0.02
– Second trimester (weeks 13–28)	14 (8.0)	47 (19.4)	11.4 (4.8 to 17.7)	<0.01
– Third trimester (weeks 29–40)	20 (10.6)	60 (24.8)	14.2 (7.0 to 21.1)	<0.01
PLPP in previous pregnancies, *n* (%)	37 (19.7)	83 (34.3)	14.6 (6.1 to 22.6)	<0.01
Duration of pain postpartum, *n* (%)				
– ⩽6 weeks	23 (62.2)	51 (61.4)	0.8 (−18.0 to 18.3)	0.93
– 7–20 weeks	8 (21.6)	18 (21.7)	(−17.2 to 14.4)	0.99
– 5–10 months	3 (8.1)	6 (7.2)	0.9 (−8.4 to 14.6)	0.86
– Persistent pain	3 (8.1)	8 (9.6)	1.5 (−12.5 to 11.3)	0.79

PLPP: pregnancy-related lumbopelvic pain; CI: confidence interval.

**Table 3.** Univariable logistic regression comparing prevalence and relevant factors (background, health, and lifestyle) between participants reporting PLPP and no PLPP by country (*n* = 979).

Factors	Italy (*n* = 481)				Norway (*n* = 435)			
	PLPP (*n* = 188) *n* (%)	No PLPP (*n* = 293) *n* (%)	Group difference (95% CI)	*p*	PLPP (*n* = 242) *n* (%)	No PLPP (*n* = 193) *n* (%)	Group difference (95% CI)	*p*
Sick leave first trimester	25 (13.3)	40 (13.7)	0.4 (−6.2 to 6.4)	0.90	18 (7.4)	9 (4.7)	2.7 (−2.1 to 7.3)	0.25
Sick leave second trimester	52 (27.7)	51 (17.4)	10.3 (2.7 to 18.1)	<0.01	40 (16.5)	13 (6.7)	9.8 (3.7 to 15.7)	<0.01
Sick leave third trimester	75 (39.9)	111 (37.9)	2.0 (−6.8 to 10.9)	0.66	102 (42.1)	48 (24.9)	17.2 (8.3 to 25.6)	<0.01
No higher education	82 (43.6)	127 (43.3)	0.3 (−8.7 to 9.3)	0.95	43 (17.8)	30 (15.5)	2.3 (−4.9 to 9.2)	0.52
Age ⩾35 (year)	93 (49. 5)	154 (52.6)	3.1 (−6.0 to 12.1)	0.50	48 (19.8)	50 (25.9)	6.1 (−1.8 to 14.1)	0.13
Multiparous	75 (39.9)	92 (31.4)	8.5 (−0.2 to 17.2)	0.06	129 (53.3)	73 (37.8)	15.5 (6.1 to 24.5)	<0.01
Prepregnancy BMI ⩾25	51 (27.1)	42 (14.3)	12.8 (5.4 to 20.4)	<0.01	70 (28.9)	51 (26.4)	2.5 (−6.0 to 10.8)	0.56
GWG above IOM^a^ guideline	40 (21.3)	38 (13.0)	8.3 (1.5 to 15.5)	0.02	66 (27.3)	26 (13.5)	13.8 (6.2 to 21.0)	<0.01
PLPP in previous pregnancies	29 (15.4)	7 (2.4)	13.0 (7.9 to 19.0)	<0.01	75 (31.0)	7 (3.6)	27.4 (20.8 to 33.8)	<0.01
Stand/walk at work	18 (9.6)	30 (10.2)	0.6 (−5.3 to 5.9)	0.83	52 (21.5)	45 (23.3)	1.8 (−6.0 to 9.8)	0.65
Perceive work as demanding	28 (14.9)	48 (16.4)	1.5 (−5.4 to 7.9)	0.66	25 (10.3)	13 (6.7)	3.6 (−1.9 to 8.8)	0.19
Regular exercise/sport^b^ prepregnancy	68 (36.2)	128 (43.7)	7.5 (−1.5 to 16.2)	0.10	177 (73.1)	139 (72.0)	1.1 (−7.2 to 9.6)	0.80
Regular exercise/sport^b^ at present (third trimester)	23 (12.2)	66 (22.5)	10.3 (3.3 to 16.8)	<0.01	73 (30.2)	80 (41.5)	11.3 (2.3 to 20.2)	0.01

PLPP: pregnancy-related lumbopelvic pain; CI: confidence interval; BMI: body mass index; GWG: gestational weight gain; IOM: Institute of Medicine.

a Gaining above was defined as upper limit of IOM range for corresponding prepregnancy BMI group (18 kg for underweight, 16 kg for normal weight, 11.5 kg for overweight, and 9 kg for obese).

b Moderate intensity (light breathing and modest sweating) leisure-time physical activity ⩾2 times weekly.

**Table 4.** Multiple logistic regression analyses and 10 factors predicting the likelihood of reporting sick leave due to PLPP among the Norwegian participants (*n* = 102 out of 435).

aOR			
Factors	aOR	95% CI	*p*
No higher education			
– Yes	1.21	0.42 to 3.51	0.73
– No	1.0		
Multiparous			
– Yes	1.27	0.59 to 2.74	0.54
– No	1.0		
BMI ⩾25			
– Yes	2.16	0.81 to 5.98	0.12
– No	1.0		
GWG above IOM^a^ guideline			
– Yes	2.16	0.81 to 5.98	0.12
– No	1.0		
Severe PLPP at present (third trimester)			
– Yes	1.97	1.09 to 3.57	0.03
– No	1.0		
PLPP in previous pregnancies			
– Yes	6.35	2.13 to 18.97	<0.01
– No	1.0		
Stand/walk at work			
– Yes	0.71	0.31 to 1.64	0.43
– No	1.0		
Perceive work as demanding			
– Yes	1.26	0.36 to 4.39	0.71
– No	1.0		
Regular exercise/sport^b^ prepregnancy			
– Yes	0.92	0.22 to 3.90	0.91
– No	1.0		
Regular exercise/sport^b^ at present (third trimester)			
– Yes	0.46	0.23 to 0.94	0.03
– No	1.0		

PLPP: pregnancy-related lumbopelvic pain; aOR: adjusted odds ratio; CI: confidence interval; BMI: body mass index; GWG: gestational weight gain; IOM: Institute of Medicine.

a Gaining above was defined as upper limit of IOM range for corresponding prepregnancy BMI group (18 kg for underweight, 16 kg for normal weight, 11.5 kg for overweight, and 9 kg for obese).

b Moderate intensity (light breathing and modest sweating) leisure-time physical activity ⩾2 times weekly.

### Factors associated with PLPP in Italy and Norway

Table 3 also shows the comparison of other relevant factors (background, health, and lifestyle) between participants reporting PLPP and no PLPP. In both countries, women with PLPP were significantly more likely to be multiparous, have GWG above IOM guidelines, and have previous experience of PLPP than those with no PLPP. For the latter, the majority reported recovery from PLPP within 7 weeks postpartum (Italy: 62.2% and Norway: 61.4%, *p* = 0.93), and less than 10% reported persistent pain (Italy: 8.1% and Norway: 9.6%, *p* = 0.79). There were, however, some between-country differences, and our adjusted analyses (Table 5) showed that a high BMI was the strongest predictor of PLPP among the Italian women (OR = 15.96, 95% CI = 2.93–86.77, *p* < 0.01) and previous PLPP (OR = 7.59, 95% CI = 0.45 to 1.84, *p* < 0.01) among the Norwegian women. For the total population (*n* = 916), women who reported maternal exercise (⩾2 times weekly) were less likely to experience PLPP (Italy: OR = 0.33, 95% CI = 0.11–1.0, *p* = 0.05 and Norway: OR = 0.52, 95% CI = 0.28–0.97, *p* = 0.04).

**Table 5.** Multiple logistic regression analyses and six factors predicting the likelihood of reporting PLPP in current pregnancy by country (Italy: *n* = 481 and Norway: *n* = 435).

Factors	Italy			Norway		
	PLPP (*n* = 188)			PLPP (*n* = 242)		
	aOR	95% CI	*p*	aOR	95% CI	*p*
Multiparous						
– Yes	1.75	0.44 to 6.88	0.43	1.24	0.64 to 2.39	0.53
– No	1.0					
BMI ⩾25						
– Yes	15.96	2.93 to 86.77	<0.01	0.92	1.04 to 5.49	0.79
– No	1.0					
GWG above IOM^a^ guideline						
– Yes	5.02	0.86 to 29.44	0.07	2.39	1.04 to 5.49	0.04
– No	1.0					
PLPP in previous pregnancies						
– Yes	1.32	0.71 to 24.58	0.85	7.59	2.67 to 21.58	<0.01
– No	1.0					
Regular exercise/sport^b^ prepregnancy						
– Yes	0.25	0.06 to 1.01	0.05	0.92	0.46 to 1.84	0.80
– No	1.0					
Regular exercise/sport^b^ at present (third trimester)						
– Yes	0.33	0.11 to 1.01	0.05	0.52	0.28 to 0.97	0.04
– No	1.0					

PLPP: pregnancy-related lumbopelvic pain; aOR: adjusted odds ratio; CI: confidence interval; BMI: body mass index; GWG: gestational weight gain; IOM: Institute of Medicine.

a Gaining above was defined as upper limit of IOM range for corresponding prepregnancy BMI group (18 kg for underweight, 16 kg for normal weight, 11.5 kg for overweight, and 9 kg for obese).

b Moderate intensity (light breathing and modest sweating) leisure-time physical activity ⩾2 times weekly.

## Discussion

This study aimed to examine the prevalence and severity of PLPP, explore self-reported sick leave, and investigate potential risk factors associated with PLPP using the same questionnaire and collected information from pregnant women in Italy and Norway. Although the overall prevalence and severity of PLPP were found to be higher among Norwegian women, Italian women also commonly reported experiencing PLPP (Italy: 39% versus Norway: 57%). The presence of PLPP during the later stages of pregnancy was found to be associated with self-reported sick leave in Norway, but not in Italy. In both countries, women with PLPP were more likely to be multiparous, have GWG above IOM guidelines, and have a previous history of PLPP compared with those without PLPP. A high BMI was the strongest predictor of PLPP among Italian women, while previous PLPP was the strongest predictor among Norwegian women. Maternal exercise was inversely associated with PLPP in both countries.

### Interpretation of findings and comparison with previous research

Based on studies from different countries,^9–13,31,32^ the prevalence of PLPP during pregnancy has been reported to range from 4% to 86%. This wide statistical range can be attributed to the use of different definitions and diagnostic methods (self-reported questionnaires, pain drawings, or clinical examinations), as well as the inclusion or exclusion of women with co-existing LBP.^2,24,33^ The prevalence of PLPP can also vary depending on the characteristics of the study population, such as gestational age and parity.^13^ For instance, multiparous women may be at higher risk than nulliparous women.^7^ This study revealed a significantly higher prevalence of PLPP among Norwegian than among Italian women, and we found some discrepancies between the participants regarding parity, with fewer Italians being multiparous. This could be one explanation for the higher proportion of women reporting PLPP in Norwegian compared with Italian women. Considering that PLPP was self-reported in this study, it is not possible to establish whether the participants suffered solely from PGP, LBP, or a combination of both. This limitation aligns with other studies where accurately distinguishing between these complaints proves difficult.^2,13^

PLPP can manifest in different degrees of severity, from a minor to a significant maternal complaint that can limit a woman’s ability to perform daily tasks and have an impact on both her physical and psychological well-being.^8^ Studies utilizing qualitative research methods have documented the emotional impact of PLPP on women and the significant challenge of managing pain while adapting and balancing everyday life.^8,21,34,35^ Severe pain occurs in about 25%, and severe disability in about 8% of women with clinically verified PLPP or PGP.^2,10,13,31^ In our study, we observed a higher level of disability among Norwegian women compared with Italian women, defined as needing to use crutches sometimes to most of the day. This difference in disability levels could be due to lifestyle factors, such as the higher levels of physical activity/exercise, including walking, among women in Norway. Also, cultural differences in coping mechanisms for pain-provoking activities may play a role in the variation in prevalence rates observed between the two countries. Furthermore, our study revealed that the Norwegian women had a higher prepregnancy BMI and GWG than the Italian women, factors that have been associated with PLPP.^7,36–38^ Unfortunately, we did not gather data on the women’s personal experience of how PLPP affected their daily activities, an aspect that has been investigated in previous studies.^8,39^

As expected, self-reported sick leave increased for each trimester, with the highest occurrence of sick leave and PLPP in the third trimester of pregnancy in both Italy and Norway. Although the frequency of self-reported sick leave was similar in both countries, we found no association between PLPP and sick leave among Italian women in late pregnancy. Nevertheless, the need for sick leave due to PLPP may be obscured by the broader category of general maternal complaints, that is, sleep problems, nausea, fatigue, pre-existing medical conditions, pregnancy complications such as hypertension and pre-eclampsia, and mental health issues.^40^ The high prevalence of sick leave due to PLPP in Norway is comparable to other studies^19,40,41^ and can be attributed to various factors that are not exclusive to Norway, but apply to the entire Scandinavia. These factors include a high level of awareness and generous sick leave policies offered by the social welfare system, the prioritization of pregnant individuals’ well-being by employers, and lifestyle factors unique to these countries.^16,18,19^ Also, the variation in employment patterns, with Norway having a notably higher percentage working at least 50% of their time away from home compared with Italy, is likely to have impacted the sick leave statistics due to PLPP.^42,43^ Finally, the numbers reporting severe PLPP were significantly higher in the Norwegian sample compared with the Italian participants. Women on sick leave also reported more severe disability compared with women not being on sick leave, thus affirming the adequacy of prescribing sick leave. Thus, future studies need to focus on effective strategies to help pregnant women manage their symptoms and continue working if possible.

Most women recover spontaneously from PLPP shortly after delivery; however, there are cases where the pain persists and becomes a long-lasting disabling condition.^38,44^ Our study’s findings are consistent with previous research showing that a small percentage of women (around 7%–10%) experience persistent pain.^2,44–46^ PLPP can have a significant impact on a woman’s quality of life and ability to return to work after childbirth, early intervention and treatment are important to prevent PLPP from becoming persistent. Health practitioners should, therefore, be mindful of the risk factors for PLPP and screen for symptoms during prenatal visits.^2,47^

### Context and underlying factors that may explain the observed results

We recruited participants from both public hospitals and antenatal clinics for this study. A previous study on Polish and Norwegian pregnant women found a PLPP prevalence rate of 42% and 56%, respectively.^24^ Interestingly, our findings closely mirrored those of Starzec et al.,^24^ who conducted a study encompassing not only health care settings but also women attending fitness clubs and yoga studios. It is worth considering that participants recruited from health care settings might exhibit a higher prevalence of PLPP compared with women drawn from the general population, especially those who participate in recreational exercise. We initially hypothesized that exercise before pregnancy would have a protective effect on PLPP. However, our findings revealed that women who exercised before pregnancy were just as likely to experience PLPP as those who did not exercise, indicating that PLPP influences exercise habits and not vice versa. This is further supported by our finding that women with PLPP were significantly less active during the third trimester compared with women reporting no PLPP. It is, however, important to acknowledge that cross-sectional studies can only identify associations between variables and not causal inference.

Some studies have reported a higher prevalence of PLPP among women with lower educational levels.^7,48^ However, our study found no significant association between education and PLPP or PLPP-related sick leave. Notably, a substantial proportion of the women in our study had completed college or university education, with a higher proportion compared with the general female population in Italy (56% versus 33%) and Norway (83% versus 40%).^42,43^ Higher educational attainment may imply less physically demanding work conditions, but it may also lead to more prolonged sitting, resulting in increased pressure and load on the pelvic region, and a fixed position of the pelvis, potentially contributing to pain.^12,49^ In both countries, PLPP was associated with previous PLPP, but not demanding working conditions, such as standing/walking, with the latter being an unexpected find and in contrast to other studies in this field.^19,48,50–52^ The discrepancy in study findings may in part be explained by differences in assessment methods and questions asked. In this study, only those reported to perform their work standing and/or walking >50% of the time were defined as having a standing posture at work.

In our study, we limited the questionnaire to women between weeks 32 and 36 of pregnancy. After this period, the uterus and fetus descend into the lower pelvis, potentially causing pain due to pressure from the fetus’ head on structures within the pelvis, which may differ from the mechanism of PLPP.^53^ We believe that this specific range of gestational weeks provides a reliable assessment of PLPP prevalence at late gestation. In the literature, it is a mix of prospective, retrospective, and cross-sectional studies, and the gestational range is often broad (weeks 12 to 41), making the prevalence numbers difficult to compare.^24,45,54–57^ Furthermore, symptoms of PLPP may vary between countries due to cultural and social factors, including differences in health care accessibility, recognition, and attitudes toward the clinical management of PLPP. For instance, some countries may have a higher proportion of women who seek medical assistance for PLPP compared with others, and the issue of whether PLPP should be endured or treated as a significant public health concern leading to sick leave has been the subject of discussion.^15,16^ In Italy, statutory sick pay is usually half of the average daily wage, while in Norway, most women receive their complete salary during sick leave. In addition, Italy mandates that anyone on sick leave must be accessible for medical spot checks at their residence to confirm their inability to work.^58^ When interpreting the prevalence of PLPP in different populations, it is essential to consider these factors.

### Strengths and limitations

There are several strengths of this study that are worth noticing. First, it has a large and equally distributed sample size from two European countries, providing insight into the prevalence and severity of PLPP across different health care systems and where awareness of this condition among health practitioners may vary. Second, our study is the first to report such data among Italian pregnant women, making it a valuable contribution to the field of maternal health. Third, we used a consistent data collection method and obtained information with a standardized, cross-language validated questionnaire within a narrow timeframe (between week 32 and week 36 of gestation), which corresponds to the late stages of pregnancy when PLPP is likely to occur and become more severe. Fourth, we chose to use a paper-based questionnaire, as research has indicated that paper-based surveys tend to yield higher response rates in comparison with online surveys.^59^ Also, studies have found that participants often perceive paper-based surveys as more anonymous than online surveys, leading to a potential increase in their honesty when responding.^60^ Finally, the inclusion of data concerning demographics and personal health variables made us able to identify and quantify the associations between multiple predictor variables and PLPP, while controlling for confounding factors.

While our study benefits from a large sample size (*n* = 916), limitations are no sample size calculation and the cross-sectional nature of the questionnaire, limiting directionality and causality between PLPP and the various factors of interest. The study enrolled women in gestational weeks 32 to 36, as the symptoms of PLPP typically peak during weeks 24 to 36.^13^ To provide a comprehensive analysis, we also gathered retrospective data on PLPP in the first and second trimesters, as well as the participants’ history of PLPP. It is worth noting that while this approach is commonly used in the literature, there is a potential limitation related to the accuracy of recalling such information.^61^ This limitation may have resulted in under- or overestimation of the prevalence and severity of PLPP, and it is possible that some cases of PLPP were missed or misclassified due to recall bias.^61^ However, it should be noted that this bias affects both study groups equally. Future research should adopt a longitudinal approach to comprehensively investigate the prevalence, severity, and impact of PLPP across different gestational stages.

In our study, the Italian and Norwegian women had higher educational level than the average level in both countries, and data collection was limited to the urban district of the capital city. Hence, we cannot generalize our findings to be representative of women in Italy and Norway. Furthermore, the classification of women who reported using crutches due to PLPP as having severe PLPP may not accurately reflect their condition. The use of crutches may be recommended early on in some cases to prevent pain progression, and the clinical practice of physical therapists and general practitioners may vary. Also, our definition of persistent PLPP does not align with recent literature, which classifies persistent symptoms as pain lasting beyond 12 weeks.^62^ In addition, the question “During this pregnancy, do you experience pain in the lumbopelvic area” may not be sufficient to ensure a proper understanding of PLPP by the participants in this study. Therefore, to enhance the accuracy of reporting, more detailed questioning could have been included, such as the use of body charts and the opportunity for participants to provide information about the location, nature, and severity of their pain. This might be particularly important for the Italian participants, who may be less familiar with the term PLPP than the Norwegian women. To improve the precision of symptom reporting, future studies should consider incorporating additional explanations and visual aids, especially in populations with lower awareness. Despite this limitation, we replicated the results from a similar study,^24^ strengthening the validity of our findings. However, it is important to recognize that the methodology used in this study, which involved quantitative analysis and cross-sectional comparison between two countries, may not entirely reflect the multifaceted nature of PLPP and sick leave during pregnancy. Clinical experience has also shown that differentiating between pain in the pelvic girdle and the lumbar spine is important for targeted treatment selection.^63^ We recommend future studies to use the Pelvic Girdle Questionnaire (PGQ) for this purpose. However, it is important to note that the PGQ was not available during our data collection in 2005 (Norway), and it has not been translated into Italian to date.

## Conclusion

While both countries had similar rates of self-reported sick leave in late gestation, an association between PLPP and self-reported sick leave was observed among Norwegian women only. Some factors associated with PLPP were consistent across the two countries, including multiparity, excessive GWG, and prior history of PLPP. In Italy and Norway, health care providers should be aware of the high prevalence of PLPP and prioritize open communication with pregnant individuals. In addition, recognizing the limited evidence on effective treatments underscores the need for further research in this field.

## Supplemental Material

sj-docx-1-whe-10.1177_17455057231218197 – Supplemental material for Lumbopelvic pain and sick leave during pregnancy: A comparison of Italy and Norway Click here for additional data file. Supplemental material, sj-docx-1-whe-10.1177_17455057231218197 for Lumbopelvic pain and sick leave during pregnancy: A comparison of Italy and Norway by Lene Annette Hagen Haakstad, Maria Beatrice Benvenuti, Emilie Mass Dalhaug and Kari Bø in Women’s Health

## Declarations

**Ethics approval and consent to participate:** The Ethical Committee of the University of Rome Foro-Italico, Italy (CARD 2018/12) and the Regional Committees for Medical and Health Research Ethics, Norway (2001/S-01191/REK), approved the project and the complete data collection. All participants signed informed written consent before answering the questionnaire. All data are nonpersonally identifiable. The STROBE guidelines were followed.

**Consent for publication:** Written informed consent for publication was provided by the participants.

**Availability of data and materials:** The data that support the findings of this study are available from the corresponding author (EMD), upon reasonable request.
